# Supplementary material for: Inheritance of associative memories and acquired cellular changes in C. elegans
Source: Nat Commun. 2023 Jul 15;14:4232. doi: 10.1038/s41467-023-39804-8 (PMC10349803; doi:10.1038/s41467-023-39804-8)
Supplement: Supplementary file 1 — Supplementary Information [file 41467_2023_39804_MOESM1_ESM.pdf]

Supplementary Figures and Tables

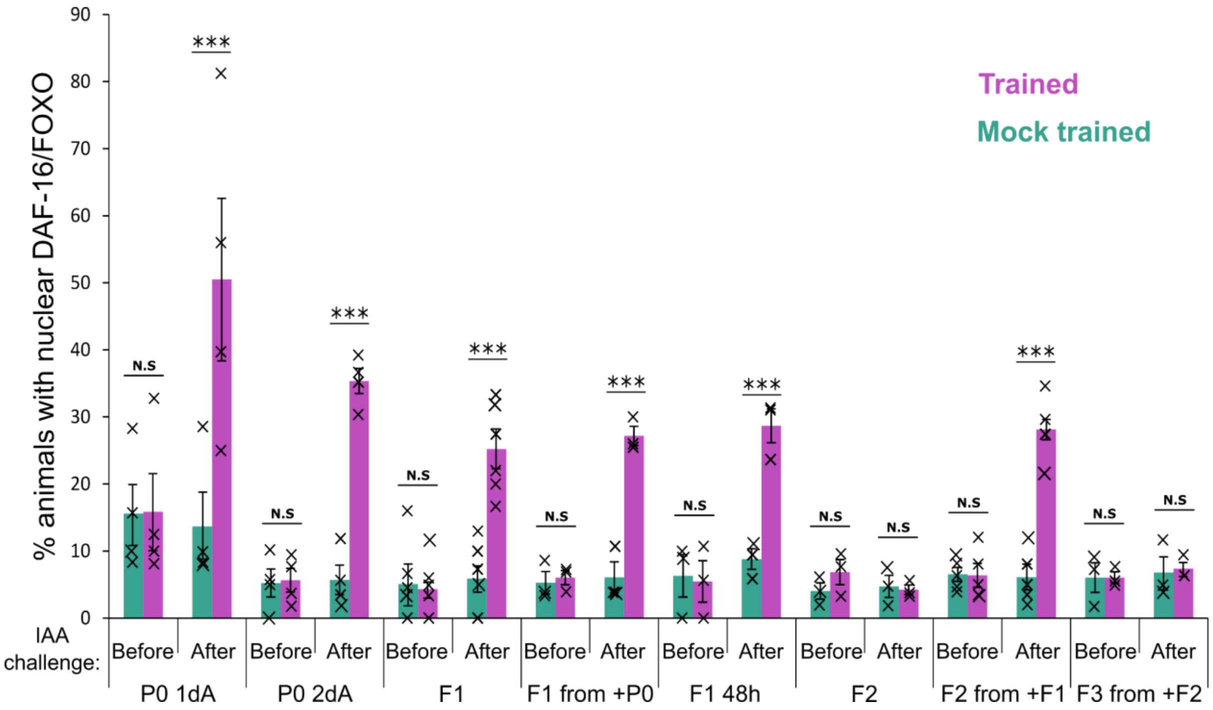

Supplementary figure 1. Acquired cellular changes are transmitted to the F1 and F2, but not to the F3, generations.

Exposure to the conditioned stimulus (CS) IAA induced the translocation of DAF-16/FOXO to cells' nuclei in the F1 and F2 generations, but not in the F3 generation. Animals were scored as having nuclear DAF-16/FOXO if at least six cells (primarily gonad sheath cells) were detected with nuclear DAF-16/FOXO within 20 minutes following exposure to the CS IAA. This cutoff is a conservative estimation and is based on our previous rigorous analysis [1]. For each generation, we scored trained and mock-trained animal groups, where each group was scored before and after challenging the animals with the CS IAA. The non-challenged groups served as a control to assess the overall stress in the population without exposure to IAA for both trained and mock-trained animals. These data provide the raw values from which figure 3C was compiled: For each group, we subtracted the % of worms with nuclear DAF-16/FOXO before the challenge with IAA from the % of worms with nuclear DAF-16/FOXO after the challenge with IAA. This subtraction was performed independently for each experimental repeat. The same subtraction procedure was used in all figures throughout the manuscript (thereafter presented as Δ%). Due to this subtraction, negative Δ% values could be obtained in cases of low % after challenge, which essentially translate to no (zero) change. +P0/+F1/+F2 denote that the assayed animals are descendants of animals (from the indicated +generation) that showed odor-evoked nuclear translocation of DAF-16/FOXO. Thus, we pre-

selected positive animals and tested their progeny. Otherwise, animals were not preselected but were randomly collected from the population. 1dA and 2dA are 1 and 2 days old adults, accordingly. 48h denotes that the assayed F1s hatched 48 hrs post the recovery of the trained P0 animals. Otherwise, all the F1 assays were performed on animals that hatched 24 h following recovery of the P0 trained animals. Each bar is the average of N=3-10 biologically independent experimental repeats, each scoring ~50 animals. Error bars indicate SEM. N.S, not significant. ***p< 0.0001 (two-sided proportion test, after Bonferroni correction). Source data is provided as a Source Data file.

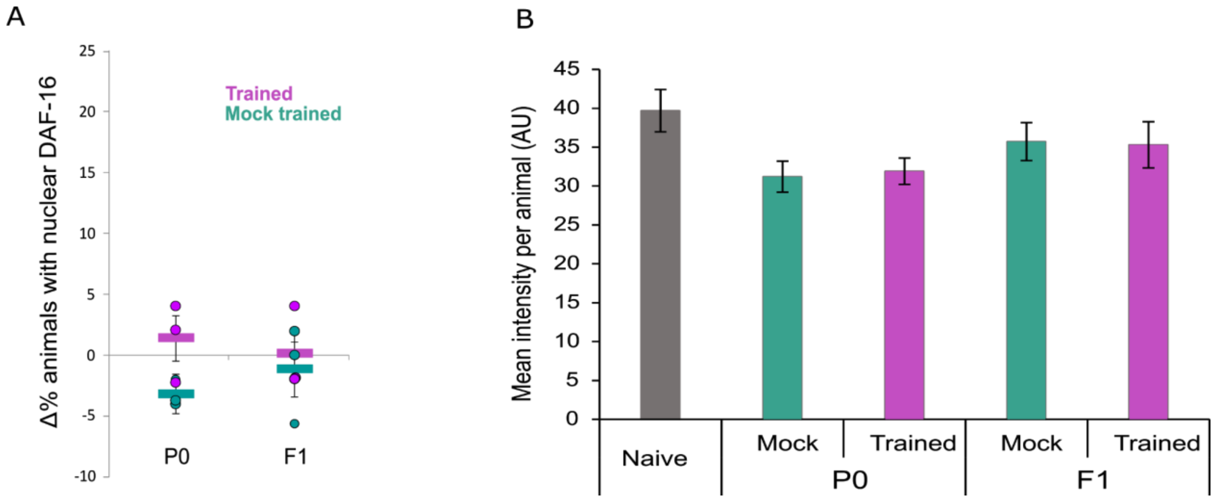

Supplementary figure 2. Worms trained to associate IAA with food do not show IAA-induced stress responses, and starvation itself does not increase DAF-16 expression levels.

(A) P0-generation worms were trained to associate IAA with food. The F1-generation animals were grown on food and were never exposed to IAA. Re-exposure to IAA did not induce a stress response in the trained P0 generation, nor in their F1 progeny, as we did not observe DAF-16/FOXO translocation to cells' nuclei. Each value is the mean of three independent experimental repeats (circles), each scoring ~50 animals. Error bars indicate SEM. P-values (P0) 0.1, (F1) 0.4 (two-sided proportion test, after Bonferroni correction).

(B) Expression levels of DAF-16 were not increased due to the repeated starvations. Expression levels were quantified based on the fluorescence levels of the DAF-16::GFP fusion protein extracted from individual animals (see Methods). Values of each animal group is the mean of 60-70 animals. Error bars denote SD. Source data is provided as a Source Data file.

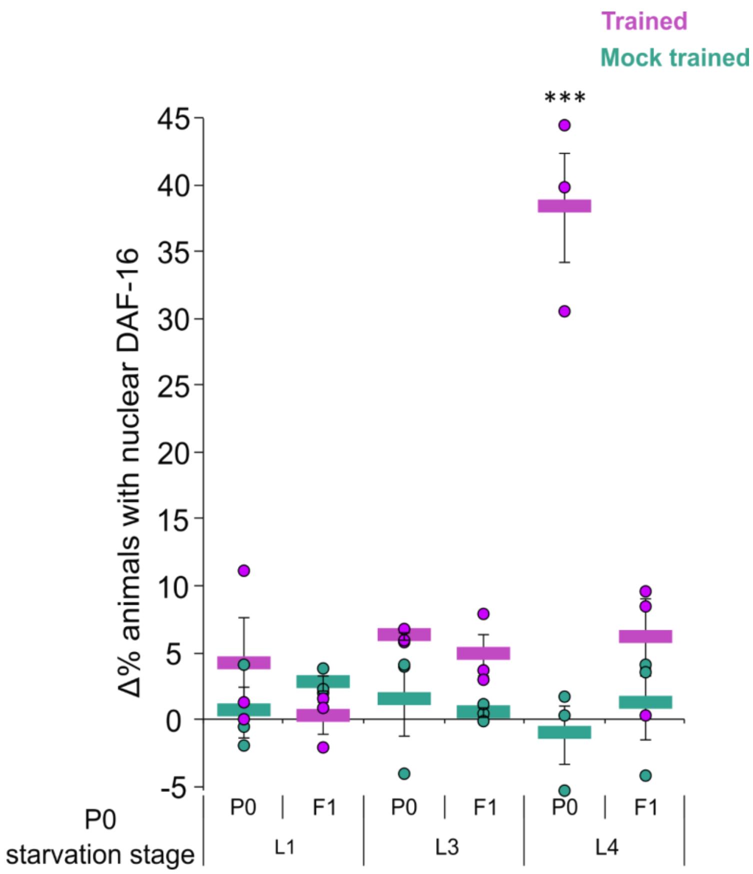

Supplementary figure 3. Training the animals in selected larval stages does not lead to inheritance of the cellular changes.

Animals were trained to associate IAA with starvation either in the L1, L3, or the L4 larval stages. The trained P0 generation and their F1 descendants were challenged with the CS IAA during their 1-day adult stage. The F1 progeny of the trained P0 animals were grown on food and never experienced starvation, nor were they exposed to IAA. Training the P0 generation in their 4th larval stage formed robust cellular changes in the P0 generation, in agreement with [1], but not in their F1 descendants. Each value is the mean of three independent experimental repeats (circles), each scoring >50 animals. Error bars indicate SEM. Significance comparisons are between trained and mock-trained animals. ***p<0.0001. P-values from left to right: 0.2, 0.3, 0.06, 0.1,1.8E-18, 0.07 (two-sided proportion test, after Bonferroni correction). Source data is provided as a Source Data file.

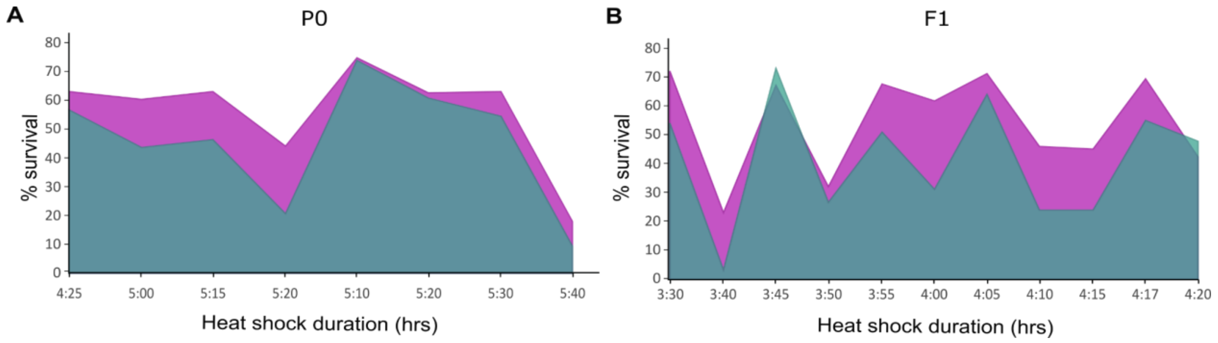

Supplementary figure 4. Exposure to the CS IAA to initiate stress responses enables both the trained P0 animals and their F1 descendants to better cope with an upcoming stress.

Survival rates of trained P0 (A) and their F1 descendants (B) were significantly higher than their mock-trained counterparts when facing heat stress following IAA exposure. Shown are the raw data used in figure 5. Odor-evoked animals were incubated in elevated temperatures to inflict the heat stress. Since the % death greatly varied across the different experimental days, incubation periods varied in their duration. However, within the same experimental day, we always compared survival rates between the same incubation times. Regardless of the exact incubation time, trained animals (and progeny of trained animals) had higher survival chances compared to their mock-trained counterparts. Purple, trained; Cyan, mock trained. (A) N=8 (B) N=14 biologically independent experimental repeats. Source data is provided as a Source Data file.

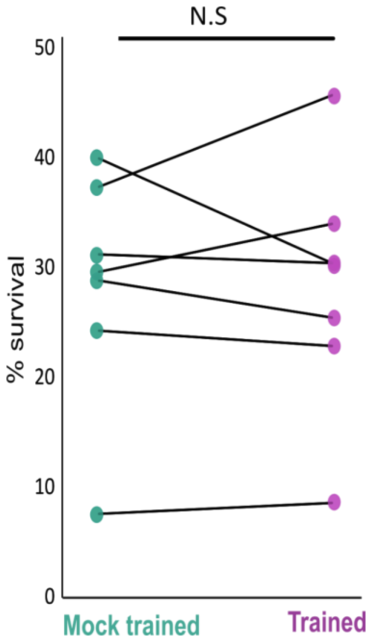

Supplementary figure 5. Survival chances were not improved by IAA alone.

Training with the conditioned stimulus IAA in the presence of food and without inflicting starvation did not increase survival rates upon exposure to IAA followed by a heat shock. Trained animals in this case refer to animals exposed to IAA on food, while mock-trained animals were not exposed to IAA while on food (essentially naive animals). Exposure to IAA was according to the spaced-training paradigm shown in figure 1A. Shown are the results for the P0 generation. Each connected pair of points represents one experimental repeat in which ~50 trained and ~50 mock-trained animals were scored. N=7, p= 0.47 (one-sided, paired t-test, Bonferroni correction). Source data is provided as a Source Data file.

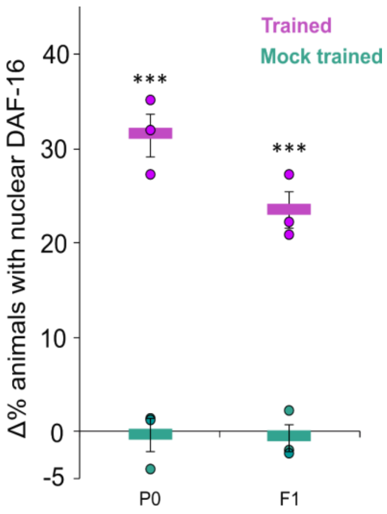

Supplementary figure 6. Histamine by itself does not affect odor-induced stress responses.

Histamine was added to trained animals that do not express the histamine-gated chloride channel. Shown are the means±SEM of three independent experimental repeats (circles), each scoring ~50 animals. Significance comparisons are between trained and mock-trained animals in each generation. ***p<0.0005. P0=9.8E-14, F1=8.0E-9 (proportion test, two-sided, after Bonferroni correction). Source data is provided as a Source Data file.

Supplementary Table 1. Reagents and resources used in this study.

REAGENT or RESOURCE	SOURCE	IDENTIFIER
**Chemicals**		
Histamine dihydrochloride	Sigma-Aldrich	CAT#H7250
Serotonin creatinine sulfate monohydrate	Sigma-Aldrich	CAT#H7752
Isoamyl alcohol	Sigma-Aldrich	CAT# W205702
Streptomycin	Sigma-Aldrich	CAT# S6501
Ampicillin	Sigma-Aldrich	CAT#A9518
**Bacterial Strains**		
E. coli: OP50	Caenorhabditis Genetics Center	RRID: WB-STRAIN:OP50
E. coli: OP50-1; Streptomycin resistant	Caenorhabditis Genetics Center	RRID: WB-STRAIN:OP50-1
C. elegans **strains**		
C. elegans wild type	Caenorhabditis Genetics Center	RRID: WB-STRAIN:N2
TJ356: *zls356* [*daf-16p*::DAF-16a/b::GFP + rol-6(*su1006*)]	[2]	RRID: WB-STRAIN:TJ356
him-5 (e1490) x TJ356	[1]	ZAS204

ZAS53 (*azrEx*53[*str-2*::ChR2-mcherry], *lite-1(ce314)*) x TJ356	[1]	ZAS170
ZAS105(*azrEx105*[*srsx-3*::ChR2-cherry+ PHA-1]; *pha-1(e2123ts)*; *lite-1(ce314)*) x TJ356	[1]	ZAS234
ZAS253 (*azrEx253*[*srsx-3*::HisCl1::SL2::NLS-mCherry + PHA-1], *pha-1(e2123ts)*, *him-5 (e1490)*) x TJ356	[1]	ZAS270
zIs356 [*daf-16p*::DAF-16a/b::GFP + *rol-6(su1006)*] x *unc-31(e928)*;	[1]	ZAS178
INV60006 (*lite-1(ce314)*; Ex[*ptph-1::Chr2-mCherry punc-122::GFP*]) x TJ356	[3]	ZAS309
ZAS204 x OH10690 (*otIs356* [*rab-3p(prom1)*::2xNLS::TagRFP]) [4]	This study	ZAS394
set-25(n5021)	Caenorhabditis Genetics Center	
set-25(n5021) x TJ356	This study	ZAS313
met-1(n4337)	Caenorhabditis Genetics Center	
met-1(n4337) x TJ356	This study	ZAS316
met-2(n4256)	Caenorhabditis Genetics Center	

met-2(n4256) x TJ356	This study	ZAS318
hpl-2(tm1489)	Mitani lab through the National Bio-Resource Project of the MEXT, Japan.	
hpl-2(tm1489) x TJ356	This study	ZAS284
wdr-5.1(ok1417)	Caenorhabditis Genetics Center	
wdr-5.1(ok1417) x TJ356	This study	ZAS328
nrde-2(gg91)	Caenorhabditis Genetics Center	
nrde-2(gg91) x TJ356	This study	ZAS420
hrde-1(tm1200)	Mitani lab through the National Bio-Resource Project of the MEXT, Japan.	
hrde-1(tm1200) x TJ356	This study	ZAS339
nrde-3(tm1116)	Mitani lab through the National Bio-Resource Project of the MEXT, Japan.	
nrde-3(tm1116) x TJ356	This study	ZAS340

egl-3(n150)	Caenorhabditis Genetics Center	
egl-3(n150) x TJ356	This study	ZAS182
CF1934: *daf-16*(mu86) I; *muIs109[daf-16p*::GFP::DAF-16 + *odr-1p*::RFP]	[5]	
**Software**		
R Project for Statistical Computing	The R Foundation	RRID:SCR_001905
RStudio Version 1.1.419	RStudio Team (2015)	RRID:SCR_000432
ImageJ	Fiji	RRID:SCR_003070

Supplementary references

1. Eliezer, Y. *et al.* A Memory Circuit for Coping with Impending Adversity. *Current Biology* **29**, 1573-1583.e4 (2019).
2. Henderson, S. T. & Johnson, T. E. daf-16 integrates developmental and environmental inputs to mediate aging in the nematode Caenorhabditis elegans. *Current Biology* **11**, 1975–1980 (2001).
3. Iwanir, S. *et al.* Serotonin promotes exploitation in complex environments by accelerating decision-making. *BMC Biology* **14**, 9 (2016).
4. Fry, A. L., Laboy, J. T., Huang, H., Hart, A. C. & Norman, K. R. A Conserved GEF for Rho-Family GTPases Acts in an EGF Signaling Pathway to Promote Sleep-like Quiescence in Caenorhabditis elegans. *Genetics* **202**, 1153–1166 (2016).
5. Kumsta, C. & Hansen, M. C. elegans rrf-1 Mutations Maintain RNAi Efficiency in the Soma in Addition to the Germline. *PLOS ONE* **7**, e35428 (2012).
